# Spatiotemporal Trends in Three Smoothed Overdose Death Rates in US Counties, 2012–2020

**DOI:** 10.5888/pcd20.220316

**Published:** 2023-03-23

**Authors:** McKinley E. Saunders, Jamie L. Humphrey, Barrot H. Lambdin

**Affiliations:** 1 Center for Health, Analytics, Media, and Policy, RTI International, Research Triangle Park, North Carolina; 2 Department of Environmental and Occupational Health, Dornsife School of Public Health, Drexel University, Philadelphia, Pennsylvania; 3 Community Health and Implementation Research Program, RTI International, Research Triangle Park, North Carolina; 4 Department of Epidemiology and Biostatistics, University of California, San Francisco; 5 Department of Global Health, University of Washington, Seattle

**Figure:**
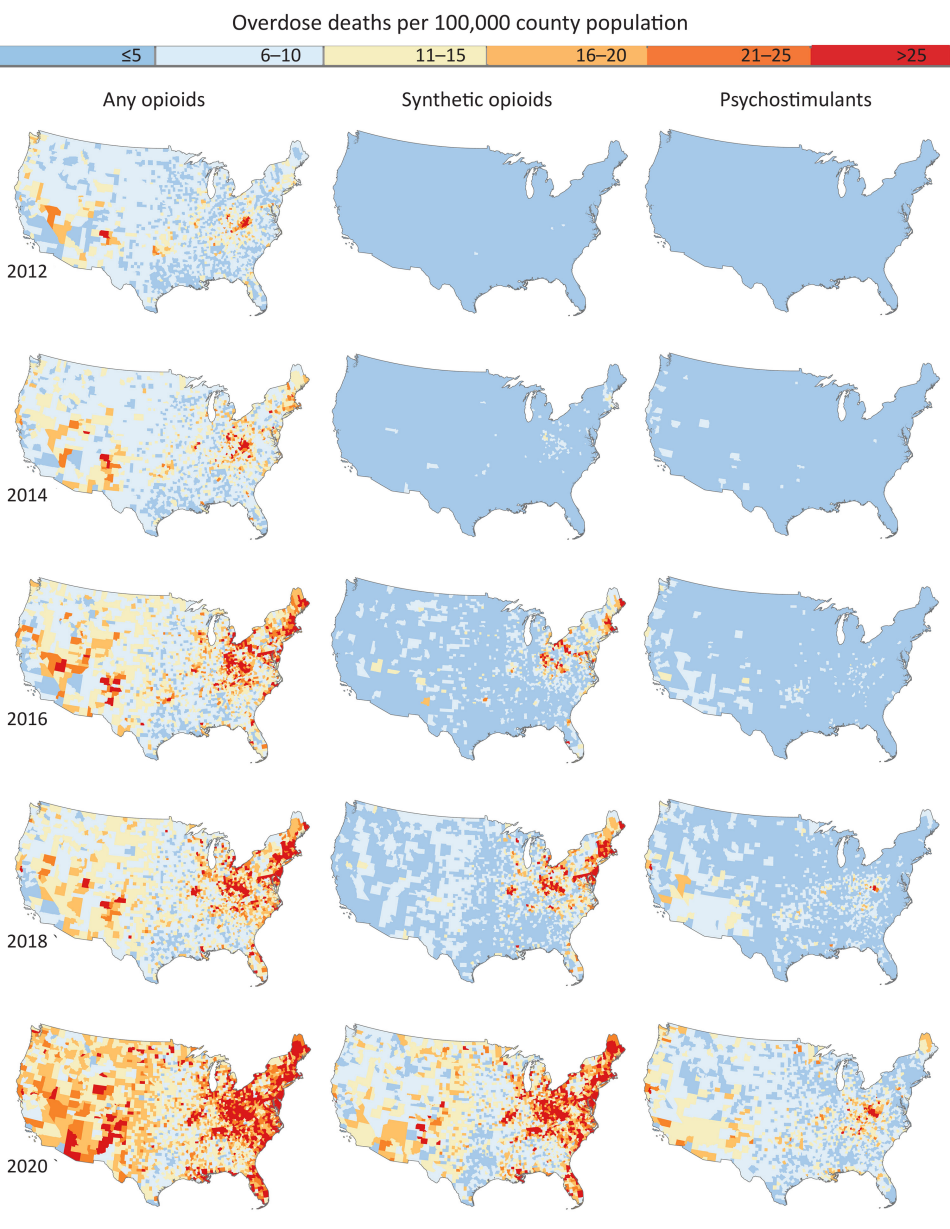
Spatiotemporal trends in smoothed overdose death rates (any opioid, synthetic opioids, and psychostimulants) in US counties (N = 3,107), 2012–2020. Mortality rates were calculated by using restricted county-level data from the National Vital Statistics System; rates were mapped for 2012, 2014, 2016, 2018, and 2020.

## Background

From 2019 to 2020, the number of drug overdose deaths in the US increased by 31%. During the same period, the rate of drug overdose deaths involving synthetic opioids other than methadone, including illicitly manufactured fentanyl, fentanyl analogs, and tramadol, increased by 56% (1). The number of overdose deaths involving psychostimulants (mostly methamphetamine) has also risen, with a 5-fold increase in these deaths from 2012 to 2018 (2).

The rise in opioid overdose deaths has occurred in 4 distinct waves (1–3). The first wave began in the 1990s and peaked around 2017 with the increase in overdose deaths involving prescription opioids (ie, natural opioids, semisynthetic opioids, and methadone). The second wave began in 2010 with increases in overdose deaths involving heroin, peaking in 2017. The third wave of overdose deaths began in 2013 with the rise in synthetic opioids (mainly illicitly produced fentanyl and fentanyl analogs) added to the heroin supply. Data suggest that we are now entering a fourth wave of overdose deaths characterized by psychostimulants with abuse potential (hereinafter, psychostimulants), which includes methamphetamine (2). Throughout these waves, the changing toxicity of the illicit drug market has influenced the geographic spread of overdose deaths (2,4,5).

We used geospatial techniques to explore county-level spatiotemporal patterns of overdose death rates during the third and fourth waves of the opioid epidemic in the contiguous US from 2012 to 2020. We describe and visualize the results of spatial empirical Bayesian smoothed overdose death rates for 3 types of overdoses. County-level spatiotemporal patterns in synthetic opioid and psychostimulant–related overdose deaths have not been systematically visualized together but are potentially useful to public health officials and researchers seeking to improve health and identify potential areas of concern.

## Data and Methods

To obtain counts of US drug overdose deaths, we used the 2012–2020 restricted-use, county-level, multiple cause-of-death files from the National Vital Statistics System (NVSS) (6). We included deaths across the entire age spectrum (<1 year to ≥85 years) and calculated death rates per 100,000 population. NVSS uses the *International Classification of Diseases, Tenth Revision* (ICD-10) (7) to code death certificate data. Drug overdose deaths were identified by using record-axis codes X40–44 (unintentional), X60–64 (suicide), X85 (homicide), or Y10–Y14 (undetermined intent). Among deaths with drug overdose as the underlying cause, we identified the type of drug or drug category as indicated by the following ICD-10 multiple cause-of-death codes: any opioid (T40.0, T40.1, T40.2, T40.3, T40.4, T40.6), synthetic opioids other than methadone (T40.4), and psychostimulants with abuse potential (T43.6) (8). Categories are not mutually exclusive; some deaths involved more than 1 type of drug and were included in rates for each drug category. We also obtained vintage postcensal bridged race population data for 2012 to 2020 from NVSS, which we used as the denominator for death rate calculations (9).

We joined the NVSS death and population data to the 2020 Census Bureau cartographic county boundaries (N = 3,107 counties) (10,11). Rates have intrinsic variance instability, which may lead to identification of spurious outliers. To correct for this, we created a first-order queen’s contiguity spatial matrix and used empirical Bayesian spatial smoothing, which improves the precision of the crude death rates by borrowing strength from other observations. The empirical Bayes technique in GeoDa version 1.18.0 (12) consists of computing a weighted average between the raw rate for each county and the national average, with weights proportional to the underlying population. For example, the rates of small counties with small populations would be adjusted considerably, whereas the rates of larger counties would hardly change (12–14). This spatial smoothing technique allows us to estimate county-level rates even in counties with small sample sizes of deaths. We mapped all smoothed overdose death rates by using ArcGIS Pro version 2.8.3 (Esri). Study procedures were reviewed and approved by the institutional review board at RTI International.

## Highlights

We observed geographic patterns emerge over time across the 3 groups of overdose deaths, consistent with previous studies (2–4). Synthetic opioid overdose deaths from 2012 to 2018 were at first regionally isolated to the Northeast and Midwest (including Appalachia) and parts of the South. However, in 2020, high synthetic opioid overdose death rates were also observed in the Mississippi River regions and the western US. 

We observed low rates of overdose deaths due to psychostimulants until 2016, similar to the pattern we found for synthetic opioids. High rates of psychostimulant overdose deaths emerged in groups of counties in the Southwest and Midwest (including Appalachia) in 2018. Then, in 2020, we observed a similar spread of high rates of psychostimulant overdose deaths throughout Appalachia and into Maine. We also found higher rates along the Gulf Coast, and in Missouri, Oklahoma, and Minnesota compared with other areas of the US. The Southwest (particularly New Mexico and Arizona), California, and Washington also had higher rates of these overdose deaths in 2020.

Any opioid-related overdose death rates were spread relatively evenly across the country in recent years, whereas overdose deaths due to synthetic opioids and psychostimulants were much higher in the Northeast and Midwest regions compared with the rest of the US. 

## Action

These maps highlight the overall pattern and geographic shifts in the US drug overdose epidemic. Whereas any opioid-overdose death rates were high across the US, drug overdose deaths consistent with the third (synthetic opioid) and fourth (psychostimulants) waves of the epidemic were higher in the Northeast and Midwest regions than in other regions. Relative to larger spatial scales, mapping county-level rates provides better granularity in describing geographic patterns of overdose deaths and improves our understanding of changes in overdose death rates over time. While it has been theorized that psychostimulant overdose deaths occurred in the same areas as synthetic opioid overdose deaths (2), these maps clearly illustrate similarities in geographic patterns of these deaths. Our findings indicate differential spatial patterning in overdose rates among different drug types, which could inform focused prevention efforts. For example, regions experiencing high or increasing levels of opioid overdose should prioritize implementation and scale-up of medications for opioid use disorder and naloxone to reverse overdoses. With methamphetamine-involved overdose deaths, little is known about the underlying mechanisms leading to death or the evidence-based interventions that could prevent them. Future research must develop and understand the effectiveness and implementation dynamics of evidence-based interventions that reduce methamphetamine-involved overdoses and associated deaths.
